# Whose body is it anyway? Cultural reflections on embodiment illusion research in eating disorders and body dysmorphic disorder

**DOI:** 10.3389/fpsyt.2024.1433596

**Published:** 2024-07-15

**Authors:** Jade Portingale, Isabel Krug, David Butler

**Affiliations:** ^1^ School of Psychological Sciences, The University of Melbourne, Melbourne, VIC, Australia; ^2^ Faculty of Psychology and Counselling, The Cairnmillar Institute, Melbourne, VIC, Australia; ^3^ Department of Psychology, Counselling and Therapy, LaTrobe University, Melbourne, VIC, Australia

**Keywords:** cross-cultural psychiatry, embodiment illusion, enfacement illusion, eating disorders, body dysmorphic disorder, body image disturbance, face perception

## Introduction

Psychological research has overwhelmingly derived from WEIRD (Western, Educated, Industrialised, Rich, Democratic) populations and a “White = neutral” perspective, neglecting cultural considerations and generalisability across diverse contexts (1, 2).

This lack of diversity is particularly evident in eating disorder (ED) and body dysmorphic disorder (BDD) research, where individuals from East/Southeast Asian countries, comprising 30% of the global population (3, 4), are underrepresented, especially those from non-WEIRD nations least culturally similar to the U.S. (e.g., Thailand, Vietnam, Japan (5);). A recent review of 377 ED studies revealed that only a small fraction of research was conducted on non-White individuals (5), and BDD research parallels this gap (6, 7). This oversight is concerning given evidence, outside the body image field, of stark cross-cultural variations in neural (8), cognitive (9), emotional, and social processing (10, 11). Moreover, Eastern countries are experiencing an increasing demand for ED treatment, highlighting the urgent need for culturally focused evidence (12).

Body image disturbance, encompassing cognitive, affective, perceptual and behavioural disturbances surrounding body shape/weight (e.g., dissatisfaction, misestimation), is integral in the onset and maintenance of EDs and BDD (13, 14). However, their manifestations diverge. EDs, such as anorexia nervosa and bulimia nervosa, are characterised by disturbed eating behaviours and attitudes driven by weight and shape concerns, typically focusing on body regions associated with adiposity (e.g., the abdomen, hips, and thighs) (15). Conversely, BDD is distinguished by a preoccupation with perceived defects in any body part, particularly the face (15–17), though the face can also be an area of concern in EDs (18). The accurate perception and representation of one’s body and face are fundamental to visual self-recognition, which is the ability to recognise one's appearance (19) and may be crucial for understanding body image disturbance in both EDs and BDD.

In this opinion piece, we highlight findings related to body and face image (hereafter ‘image’) disturbance across Western and East/Southeast Asian individuals from WEIRD and non-WEIRD countries. We emphasise the crucial need to consider cultural factors when generalising image disturbance research, particularly to non-WEIRD East/Southeast Asian populations. Embodiment illusions (e.g., experiencing ownership over a fake rubber hand (20, 21)) could provide insights into the mechanisms underlying image disturbances and potential ED and BDD treatment avenues. However, exploring cultural differences and considerations in their application to East/Southeast Asian (and other non-WEIRD) populations is necessary.

## Cross-cultural differences in body and face image disturbance

ED and BDD research predominantly in WEIRD societies limits generalisability to other cultures (5, 6, 22). However, recent studies have begun to explore the prevalence and presentation of these disorders in East/Southeast Asian populations. Despite the increasing prevalence of body image disturbance in East/Southeast Asian groups, narrowing the gap with the West (22–25), when considering EDs, a recent review of 33 studies found that lifetime and 12-month ED prevalence rates were 8.5 times higher in Western countries than Asian countries (26). Research on BDD in Asian populations is limited, and studies directly comparing the prevalence of BDD cross-culturally are scarce. However, one study found that the muscle dysmorphia variant of BDD was more common in Western countries compared to East Asia (27). Understanding cross-cultural variations in image disturbances remains crucial.

Cultural standards of beauty may influence the manifestation of image disturbances in several ways. For instance, women in East/Southeast Asia have been shown to exhibit a stronger drive for thinness and a greater desire to lose weight compared to their Western counterparts (28). These differences may stem from thinness being a more longstanding ideal in East/Southeast Asian cultures versus more recent and ever-evolving Western thinness trends (29, 30), although this view is not universally accepted (31). Furthermore, limited research suggests that East/Southeast Asian populations prioritise facial features over body weight and shape in attractiveness evaluations and experience greater dissatisfaction with facial features that distinguish them from other cultures, such as the eyes and nose (32, 33). This is reflected in the higher rates of facial cosmetic surgeries (e.g., eyelid surgery) in East/Southeast Asian individuals, while Western individuals report more body-focused surgeries (e.g., liposuction (34)). However, more research is needed to confirm these potential cultural differences and researchers must exercise caution when generalising findings from WEIRD populations to Asian contexts.

Sociocultural factors, such as acculturative stress (35), may contribute to cross-cultural differences in image disturbances. However, the underlying neuro-cognitive mechanisms driving these differences remain unclear. Investigating visual self-recognition may provide valuable insights, as individuals with body image disturbances show increased errors and/or decreased accuracy in recognising their own face compared to healthy controls (15, 36). Exploring this aspect further could elucidate the role of visual self-processing in the development and maintenance of image disturbances cross-culturally.

## Cross-cultural differences in visual self-recognition?

Evidence suggests cultural differences in visual self-recognition. Children from non-WEIRD countries (e.g., Kenya, Vanuatu) have been shown to pass mirror self-recognition tasks at lower rates than White (WEIRD) children (37, 38). White participants also show faster self-face identification and stronger frontal-central brain responses than Chinese participants (39).

These disparities may stem from cultural variations in self-construal and attentional patterns (40). Independent self-construals in Western cultures may facilitate robust self-representations through self-focused attention (41). Conversely, interdependent self-construals in Eastern cultures (42) may reduce self-directed processing, impacting the robustness of facial self-representations. Additionally, traditional Asian beauty ideals emphasising specific facial features (43) may heighten sensitivity to perceived deviations from idealised facial representations, engendering facial dissatisfaction and facial appearance concern. In contrast, historical appearance standards in WEIRD cultures place less significance on specific facial features (e.g (32).,), potentially leading to more generalised body image disturbance.

Culturally modulated variations in self-focused facial processing could make East/Southeast Asian individuals more susceptible to distorted facial perception and dissatisfaction, potentially explaining heightened facial distress in these populations compared to more generalised body dissatisfaction in White samples. Understanding cultural variations in perceiving and attending to facial features is crucial for elucidating how EDs and BDD manifest across cultures. Next, we focus on the perceptual basis of self-recognition: multisensory integration.

## Multisensory integration, embodiment illusions and body image disturbance: current research

The perception of our body and face is achieved through the continuous integration of multisensory inputs (i.e., visual, tactile, proprioceptive, and interoceptive) (44, 45). Embodiment illusions provide a novel approach to investigating the multisensory integration mechanisms underlying how we build and sustain body image and potential cross-cultural differences in image disturbances. These paradigms induce illusory ownership over external bodies or body parts by introducing multisensory conflicts across modalities like vision and touch. For instance, experiencing stroking on one’s own (unseen) hand whilst observing a rubber hand being synchronously stroked typically elicits perceived ownership over the rubber hand (20, 21). Similarly, virtual reality allows inducing full-body illusions through visuo-tactile synchrony with other body parts or an entire body (46, 47). Predictive coding frameworks suggest that the brain realigns discrepant sensory inputs with internal models to maintain coherent bodily representations (44).

Researchers have increasingly utilised embodiment illusions to understand and improve perceptual body image disturbance in EDs and BDD (i.e., body shape/weight misestimation), which has been neglected compared to cognitive, affective, and behavioural components in current research and intervention (48). A recent systematic review by Portingale et al. (48) found that individuals with higher body image disturbance were more susceptible to these illusions, indicating potential multisensory integration deficits underlying perceptual image disturbances. Embodiment illusions were also shown to update and improve disturbed body perceptions: e.g., experiencing a full-body illusion with a healthy weight model reduced body size overestimation in anorexia nervosa samples. Both susceptibility and improvement effects were consistently medium to large. These findings suggest the non-trivial role of embodiment illusions in understanding the mechanisms underlying image disturbances and developing potential interventions. It is imperative to extend research beyond WEIRD cultures to ensure a comprehensive mechanistic understanding and effective treatments for diverse populations.

## Generalisability of current embodiment illusion research to East/Southeast Asian populations and future research directions

Coinciding with concerns around the broader ED and BDD literature (3, 6, 7, 49), the generalisability of embodiment illusion research to East/Southeast Asian populations (along with non-WEIRD countries) is severely limited due to underrepresentation. Of the 32 studies reviewed by Portingale et al. (48), only one included a non-WEIRD Asian sample, demonstrating that avatar embodiment improved body perceptions in Taiwanese individuals (50): however, this study did not assess illusion susceptibility or compare cultures. The remaining 31 reviewed studies from WEIRD samples also failed to address ethnicity or cultural factors.

**Figure 1:**
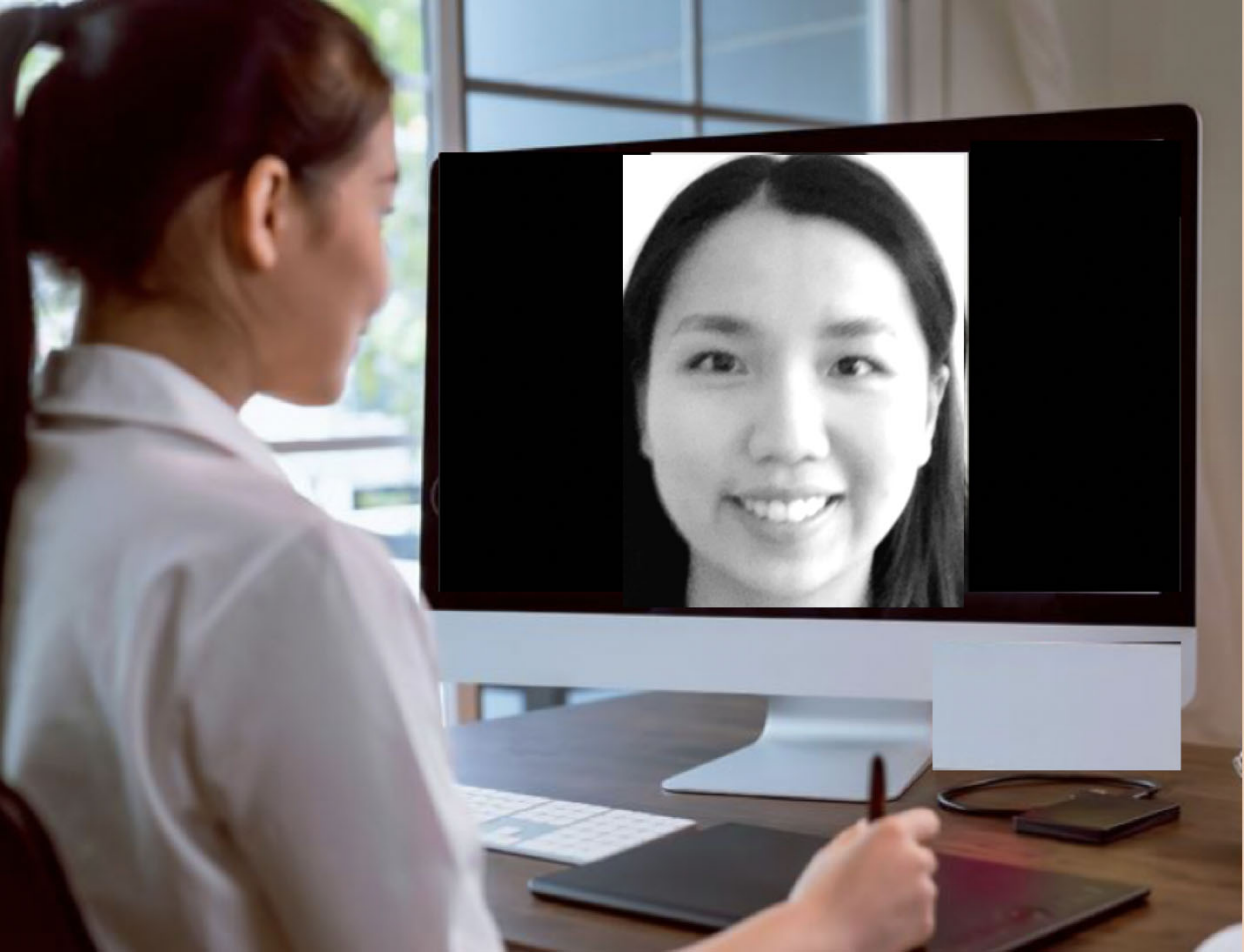
Example of the online enfacement illusion procedure. Depicted here is synchronous facial mimicry with a smiling expression (i.e., tactile-reduced stimulation).

Notably, no published study investigated susceptibility and/or improvements regarding image disturbance via the “enfacement illusion”—which induces a sense of ownership over another’s face through synchronous interpersonal multisensory stimulation (51), such as mimicking the facial expressions of an actor observed in a computer screen (**Figure 1**). Moreover, no published study included measures of self-face image when examining embodiment illusions. These oversights are critical, given evidence suggesting a heightened emphasis on facial features in body image evaluations among East/Southeast Asian populations (e.g (32, 33).,) and potential self-face recognition differences in these populations.

Further research is needed on cultural differences in image disturbances, their underlying mechanisms and treatment. First, predictive coding accounts propose that prior experiences shape top-down expectations that guide perception (44, 45). If East/Southeast Asian individuals prioritise and experience facial features differently than WEIRD populations—due to cultural variations in self-construal, attentional patterns, and beauty ideals—the magnitude of susceptibility to embodiment and enfacement illusions and nature of improvements following these illusions may differ cross-culturally. For example, East/Southeast Asian individuals may experience stronger enfacement illusions than full-body illusions, while the opposite pattern may be observed in WEIRD cultures. Moreover, enfacement illusions targeting salient facial regions like the eyes may induce more improvements in image disturbances in East/Southeast Asian individuals than full-body illusions. Examining such may elucidate potential cross-cultural differences in multisensory mechanisms underlying face and body image disturbance and allow for more targeted ED/BDD interventions.

Second, future cross-cultural studies should integrate psychological measures with biological markers during embodiment tasks. Differences in facial feature gaze patterns between East/Southeast Asian and WEIRD populations (52), possibly related to variations in self-construal, suggest potential differences in self-face perception and underlying multisensory mechanisms. Eye tracking and fMRI could elucidate cross-cultural differences in neural processes underlying illusion susceptibility and post-illusion improvements in image disturbances. Nuanced measures of face image disturbance are also needed, such as those capturing different components (e.g., dissatisfaction, concern, adiposity, avoidance, checking, contemplating surgery) and different facial features (e.g., eyes, nose) (48, 53).

Third, future research must be conducted in a culturally sensitive manner, considering the unique sociocultural contexts and values of the studied populations (54–57). Future research should also go beyond WEIRD and Asian samples to include other non-WEIRD countries (e.g., traditional societies) for a more complete understanding of the manifestation and treatment of EDs and BDD globally. Recent work has used culturally neutral methods to evaluate embodiment in EDs (58). Future studies should consider replicating and extending these methods in diverse cultural contexts, particularly non-WEIRD populations, to better understand embodiment’s role in body image disturbances cross-culturally.

Lastly, researchers should consider updating theories of body image to incorporate cultural similarities and differences. For example, Slade’s (59) schematic model of body image proposes that body image consists of several factors, including history of sensory input, biological factors, and cognitive and affective processes. While Slade acknowledged cultural/social norms as one component, many of the suggested components could be considered from a cross-cultural perspective. For instance, cross-cultural differences in exposure to self-stimuli (e.g., face vs. body; vision vs. touch) could influence the wiring of brain networks associated with self-perception, especially regarding how it integrates multisensory information typically associated with self-perception. These differences in sensory exposure and neural wiring may lead to cross-cultural differences in embodiment illusion susceptibility and/or how these illusions influence improvements in body image. Understanding how these components vary cross-culturally may provide insights into differences in susceptibility to embodiment illusions and their potential positive effects on image disturbances.

## Conclusions

The underrepresentation of East/Southeast Asian populations in embodiment illusion research limits the generalisability of findings and their potential clinical implications for EDs and BDD. Future culturally sensitive studies adapting embodiment illusions are crucial for developing and understanding effective interventions targeting potential multisensory integration disturbances underlying image disturbances in diverse populations.

## Ethics statement

Written informed consent was obtained from the individual(s) for the publication of any identifiable images or data included in this article.

## Author contributions

JP: Conceptualization, Investigation, Writing – original draft, Writing – review & editing. IK: Conceptualization, Supervision, Writing – review & editing. DB: Conceptualization, Supervision, Writing – review & editing.
